# Severe Sleep Deprivation Causes Hallucinations and a Gradual Progression Toward Psychosis With Increasing Time Awake

**DOI:** 10.3389/fpsyt.2018.00303

**Published:** 2018-07-10

**Authors:** Flavie Waters, Vivian Chiu, Amanda Atkinson, Jan Dirk Blom

**Affiliations:** ^1^Clinical Research Centre, Graylands Hospital, North Metropolitan Health Service–Mental Health, Perth, WA, Australia; ^2^School of Psychological Sciences, University of Western Australia, Perth, WA, Australia; ^3^Division of Psychiatry, University of Western Australia, Perth, WA, Australia; ^4^Parnassia Psychiatric Institute, The Hague, Netherlands; ^5^Faculty of Social and Behavioural Sciences, Leiden University, Leiden, Netherlands; ^6^Department of Psychiatry, University of Groningen, Groningen, Netherlands

**Keywords:** sleep restriction, homeostatis, hallucination, delusion, illusion, distortion, metamorphopsia, misperception

## Abstract

**Background:** Going without sleep for long periods of time can produce a range of experiences, including perceptual distortions and hallucinations. Many questions, however, remain unanswered regarding the types of symptoms which are most reliably elicited, the time of symptom onset, and whether symptoms worsen over time toward psychotic decompensation. Since sleep deprivation exceeding 48 h is considered unethical today, an examination of historical studies with extreme sleep-loss duration is needed to obtain information about what happens during prolonged sleep loss.

**Methods:** A systematic-review approach was used to identify experimental and observational studies of sleep deprivation in healthy people which describe the effects of prolonged sleep loss on psychopathological symptoms, without any date restriction.

**Results:** A total of 476 articles were identified. Of these, 21 were eligible for inclusion. Duration of sleep loss ranged between 24 h and 11 nights (total 760 participants; average 72–92 h without sleep). All studies except one reported perceptual changes, including visual distortions (i.e., metamorphopsias), illusions, somatosensory changes and, in some cases, frank hallucinations. The visual modality was the most consistently affected (in 90% of the studies), followed by the somatosensory (52%) and auditory (33%) modalities. Symptoms rapidly developed after one night without sleep, progressing in an almost fixed time-dependent way. Perceptual distortions, anxiety, irritability, depersonalization, and temporal disorientation started within 24–48 h of sleep loss, followed by complex hallucinations and disordered thinking after 48–90 h, and delusions after 72 h, after which time the clinical picture resembled that of acute psychosis or toxic delirium. By the third day without sleep, hallucinations in all three sensory modalities were reported. A period of normal sleep served to resolve psychotic symptoms in many—although not all—cases.

**Conclusions:** Psychotic symptoms develop with increasing time awake, from simple visual/somatosensory misperceptions to hallucinations and delusions, ending in a condition resembling acute psychosis. These experiences are likely to resolve after a period of sleep, although more information is required to identify factors which can contribute to the prevention of persistent symptoms.

## Introduction

Studies are reporting that we, as a society, are sleeping less and less (1). This is concerning, given evidence of the negative impact of sleep loss on health and wellbeing (2). Long periods without sleep are associated with cognitive difficulties, and can produce psychological symptoms ranging from mood changes to psychotic experiences such as hallucinations (3, 4). Given that chronic sleep difficulties affect approximately 10–30% of the population, and that for some people, sleeplessness is an occupational hazard (5, 6), it is important to better understand the psychopathological effects of sleep deprivation, and what happens with increasing time awake. This paper presents an examination of psychopathological experiences reported by healthy individuals who participated in sleep-deprivation studies ranging in duration from 24 h up to 11 days. We were particularly interested in the phenomenological description of perceptual distortions and hallucinations, and the changes that occur with progressive sleep loss.

The interaction between sleep loss and psychotic symptoms has long been known. Historical texts speak of the erstwhile practice of torturing those accused of witchcraft by depriving them of sleep, and of the psychotic states that inevitably ensued (7).

There is also an extensive clinical literature describing the link between sleep deprivation and acute psychotic states. Studies in schizophrenia and bipolar disorder show that sleep problems are among the most prominent correlates of positive symptoms—such as auditory hallucinations and delusions—and illness severity. Studies also show that many psychotic episodes are preceded, if not precipitated, by prolonged insomnia (8–12). Insomnia is a well-known clinical stressor, and it is indeed considered a prodromal symptom of psychosis (13, 14). Finally, clinical studies have observed the dynamic relationship that exists between sleep and symptoms, with reductions in sleep duration being directly followed by increases in psychotic symptom severity with a time lag of approximately 1 day (8, 15–17).

Perceptual distortions and hallucinations after a period of sleep loss have also been reported in individuals with no history of psychiatric illness. These sleep-loss phenomena offer the opportunity to study the continuum of perception in healthy humans from the point of view of a normally occurring stressor (18). One common approach to the subject of sleep loss involves epidemiological studies conducted in the general population. These show that sleep problems correlate with an increased frequency of psychotic disturbances such as hallucinations and delusional beliefs [e.g., (19–22). For instance, Sheaves et al. (21), *N* = 15,983] showed that sleep difficulties in a general-population sample were associated with a 2- to 4-fold increase in hallucination frequency. Another large study involving 45 countries demonstrated that sleep problems increase the odds of at least one psychotic symptom by 1.45 (19).

One limitation of epidemiological studies is that they are not designed to chart causal inferences, or observe subtle symptom changes which occur as a direct function of time spent awake. Moreover, epidemiological studies are not suited for capturing detailed information about symptom phenomenology. For example, it is not clear which sensory modality is most commonly affected when hallucinations arise in the context of sleep deprivation, and which other psychotic symptoms are reported. Is the symptom profile more similar to schizophrenia-spectrum disorders (with its predominance of auditory hallucinations, distorted thinking and delusions), or to hallucinations in individuals with eye disease or neurodegenerative disorders (in whom visual hallucinations are more common)?

Prospectively assessing symptom changes after sleep loss is not an easy task, as it requires detailed monitoring of time spent awake and repeated tracking of mental changes over time. Therefore, sleep-deprivation studies are ideally suited for examining the dynamic effects of sleep loss on symptoms. In sleep-deprivation studies, participants are kept continuously awake for prolonged periods of time [e.g., (3, 23)]. It is a powerful methodological tool to examine whether sleep loss is causally related to mental changes. It allows for full control over sleep duration as an independent variable, and provides optimal conditions to examine the dose-dependent relationships that might exist between the duration of time awake and symptoms.

Few studies of the impact of sleep loss on psychological symptoms have been conducted in the past 20 years [e.g., (3, 23–26)]. These studies show that going without sleep for one or two nights can induce powerful perceptual changes, but they have not been able to extend the experiments past 2 days without sleep. Extreme sleep-deprivation studies (i.e., more than two nights without sleep) were popular in the first half of the twentieth century, but are no longer conducted because they are considered unethical, and in most countries are prohibited by law. Since they cannot be performed anymore, we need to revisit early historical studies to obtain information about what happens after very prolonged sleep loss.

The present study therefore aims to describe and appraise published studies on the effects of sleep deprivation on psychopathological symptoms in healthy volunteers, without any date restriction. Using a systematic-review approach to identify relevant studies, the questions we asked were as follows:

1. Is there a causal relationship between sleep loss and perceptual distortions and/or hallucinations?
2. What sensory modality is the most commonly affected?
3. What other symptoms are elicited after sleep deprivation?
4. Do symptoms evolve as a function of increasing time spent awake?
5. Do symptoms spontaneously resolve after a period of normal sleep?

## Methods

### Search strategy

We used a systematic-review approach to identify experimental and observational studies which reported on the impact of prolonged sleep loss on psychopathological symptoms. A systematic search was carried out in Embase (which includes Medline titles) without any date restriction. The date of the last search was May, 2018. Search terms were (sleep depriv^*^ OR sleep restrict^*^) AND (psychopath^*^ OR percept^*^ OR sens^*^ OR hallucinat^*^ OR psychos^*^ OR psychot^*^ OR symptom^*^).

### Inclusion/exclusion criteria

Included were experimental studies, observational studies, and case reports, written in English, which provided (i) original data involving healthy individuals, with (ii) a sleep-deprivation or sleep-restriction design, with (iii) measurement or assessment of symptoms, and with (iv) experimenter supervision. Excluded were studies involving psychiatric populations, involuntary participants, treatment studies, drugs, reviews, and meta-analyses. Moreover, all studies that lacked symptom assessments or reports were excluded.

### Data analysis

All eligible papers were analyzed, and the following variables were extracted: year of publication, type of experimental setting, number of participants, demographic data, duration of sleep deprivation or restriction, perceptual changes and other psychological changes. An initial examination of participants' reports revealed a range of phenomena, classified and defined as follows (27):

- *Hallucination:* when perceptions lacked a corresponding stimulus in the external world (i.e., new perceptual creations),
- *Illusion:* when perceptions were based on a real stimulus, but were either misperceived or misinterpreted,
- *Distortion:* when perceptions were based on a real stimulus, but displayed changes in the intensity, quality or form of the perceived object or scene (e.g., seeing things as larger or smaller than they actually are). When such distortions occurred in the visual modality, they were referred to as *metamorphopsias*.

## Results

### Study selection

**Figure 1:**
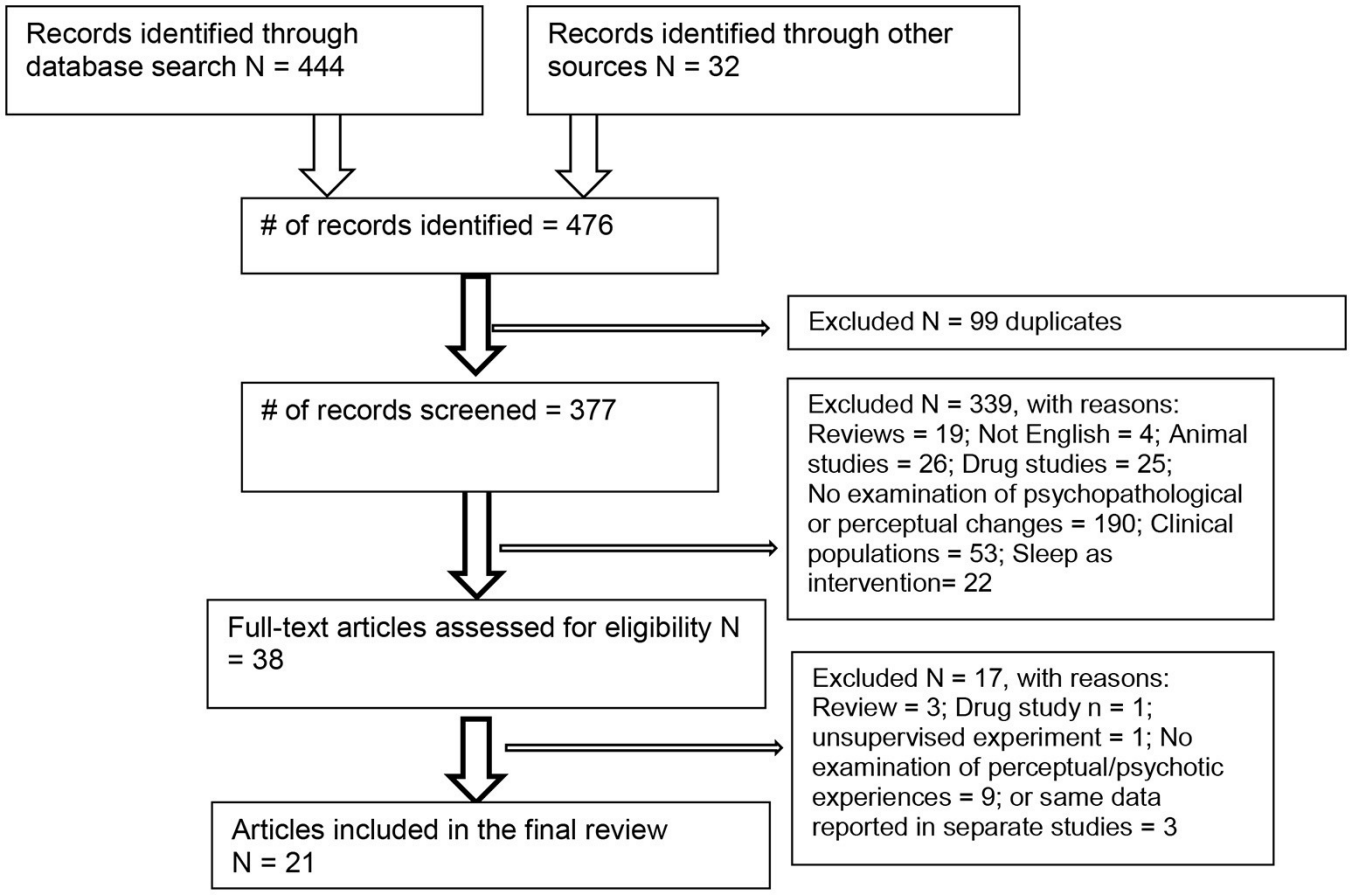
PRISMA diagram.

Figure 1 shows that the search yielded 444 articles, as well as 32 from historical sources and cross-referencing (total *N* = 476). After removal of duplicates, 377 papers remained to be examined for suitability, leaving 38 papers which were downloaded. A further 17 articles were removed (reasons given in Figure 1), leaving 21 articles which met the full criteria, reporting on 760 participants.

### Design and participant description

Participants comprised volunteers recruited from the general community, the military, and universities, as well as medical interns (Table 1). Most were male (88.8%, gender not provided for 16 participants). The mean age was 36.5 years, with a range of 17–46 years (excluding six studies without age data).

**Table 1:** List of studies included in the review and summary of key findings.

				**Hallucinations and other perceptual changes**			**Other psychopathological changes**				
**Citation**	***N***	**Setting (participant description)**	**Duration without sleep**	**% of participants positive findings**	**With Visual**	**Auditory**	**Somatosensory**	**Delusions**	**Disordered thoughts**	**Time distortions**	**Dissociation**	**Mood**
(26)	207	Sleep laboratory (military)^*^	24 h	Not reported	✓	✓	✓					✓
(25)^a^	32	Sleep laboratory (students)^*^	24 h	43%	✓	✓	✓		✓	✓		✓
(31)	14	Hospital (medical interns)^*^	32 h (6% sleep)	Nil					✓		✓	✓
(18)	10	Laboratory (N/S)	48 h (*n* = 2); 72 h (*n* = 8)	60%	✓							
(3)	25	Laboratory (military personnel)^*^	56 h	Not reported			✓	✓				
(36)	3	Laboratory (staff/students)	60 h	100%	✓	✓	✓	✓	✓			
(29)	6	Laboratory (community)	64 h−115 h (2.5–4.7 days)	Nil	✓	✓	✓		✓		✓	✓
(37)	7	Hospital (medical students)^*^	72 h (3 days)	100%	✓	✓	✓		✓	✓	✓	✓
(33)	20	Talkathon (community)^*^	88 h	100%	✓		✓			✓	✓	
(45)	3	N/S (community)	90 h	33%	✓				✓			
(35)	6	Laboratory (medical students)	96 h (4 days)	100%	✓	✓		✓	✓			
(30)	26	Research ward (military)^*^	98 h	46%	✓		✓		✓	✓	✓	✓
(34)	27	N/S (students)^§^	100 h	100%	✓	✓	✓	✓	✓	✓		✓
(32)	350	Military camp (military)^§^	112 h	11%	✓	✓		✓				✓
(48)	4	Research ward (students)^*^	120 h (5 days)	100%	✓		✓		✓		✓	✓
(39)	12	Hospital (N/S)^§^	123 h	“Some”	✓	✓		✓	✓		✓	✓
(41)	1	Radio marathon (community)^§^	168 h (7 days)	*N* = 1 (yes)	✓	✓		✓	✓	✓		✓
(42)	4	Research ward (students)^*^	205 h (8 days)	100%	✓							✓
(28)	1	Home (community)^§^	231 h (9 days, 2% sleep)	*N* = 1 (yes)	✓							✓
(40)	1	Radio marathon (community)^§^	252 h (10 days)	*N* = 1 (yes)	✓							✓
(46)	1	Laboratory (community)^§^	264 h (11 days)	*N* = 1 (yes)	✓							✓

* Study using questionnaires or symptom rating scales;

§ *Study using mental health interviews or self-reports*.

a *Petrovsky et al. (23) and Meyhöfer et al. (25) report on the same participants and only the former is reviewed here*.

A standard procedure involved participants being kept to a laboratory or other defined area, reading, watching TV, talking or playing board or card games. A team of researchers, working in shifts, attended closely to these participants, preventing them from dozing off or napping. When participants appeared drowsy, they were made to walk or engage in gentle physical activity. Validated questionnaires (*n* = 9), clinical interviews (*n* = 3), participant log records, and/or experimenter observations (cumulative sum *n* = 18) were completed at regular intervals to capture the presence of symptoms (Table 1). Fifteen studies were conducted in hospital conditions, with the detailing of neurological or psychological assessments. Three studies were conducted at a radio studio, and another one in the participant's home (Table 1).

Across the 21 articles, the duration of sleep deprivation ranged from 24 h (one night) to 264 h (11 nights). The most common duration was three or four nights without sleep (72–96 h, in 45% of the studies). The duration of sleep deprivation was usually predetermined, except for four studies in which the participants were asked to stay awake as long as they could (18, 28–30).

In two studies, very brief episodes of intermittent sleep had been allowed (28, 31). In the study involving medical interns (31), participants slept for 2 h during a 32-h period (i.e., 6% of the total duration of sleep loss). Katz and Landis' participant slept for 4 h (i.e., 2% of the total period).

### Is there a causal relationship between sleep loss and perceptual distortions and/or hallucinations?

Table 1 provides an overview of experiences reported during the experiments. Perceptual distortions and hallucinations were reliably elicited by a majority of participants in all studies except one (20/21 studies, 95%). In larger samples (where percentages are more meaningful), percentages of positive responses ranged between 11% [(32), *N* = 350], 43% [(25), *N* = 32], 46% [(30), *N* = 26], 60% [(18), *N* = 10] and 100% [(33), *N* = 20; (34), *N* = 27]. The only study which failed to register any changes in perception involved medical interns who were allowed a brief sleep period of 4 h (31).

### What sensory modality is the most commonly affected?

**Figure 2:**
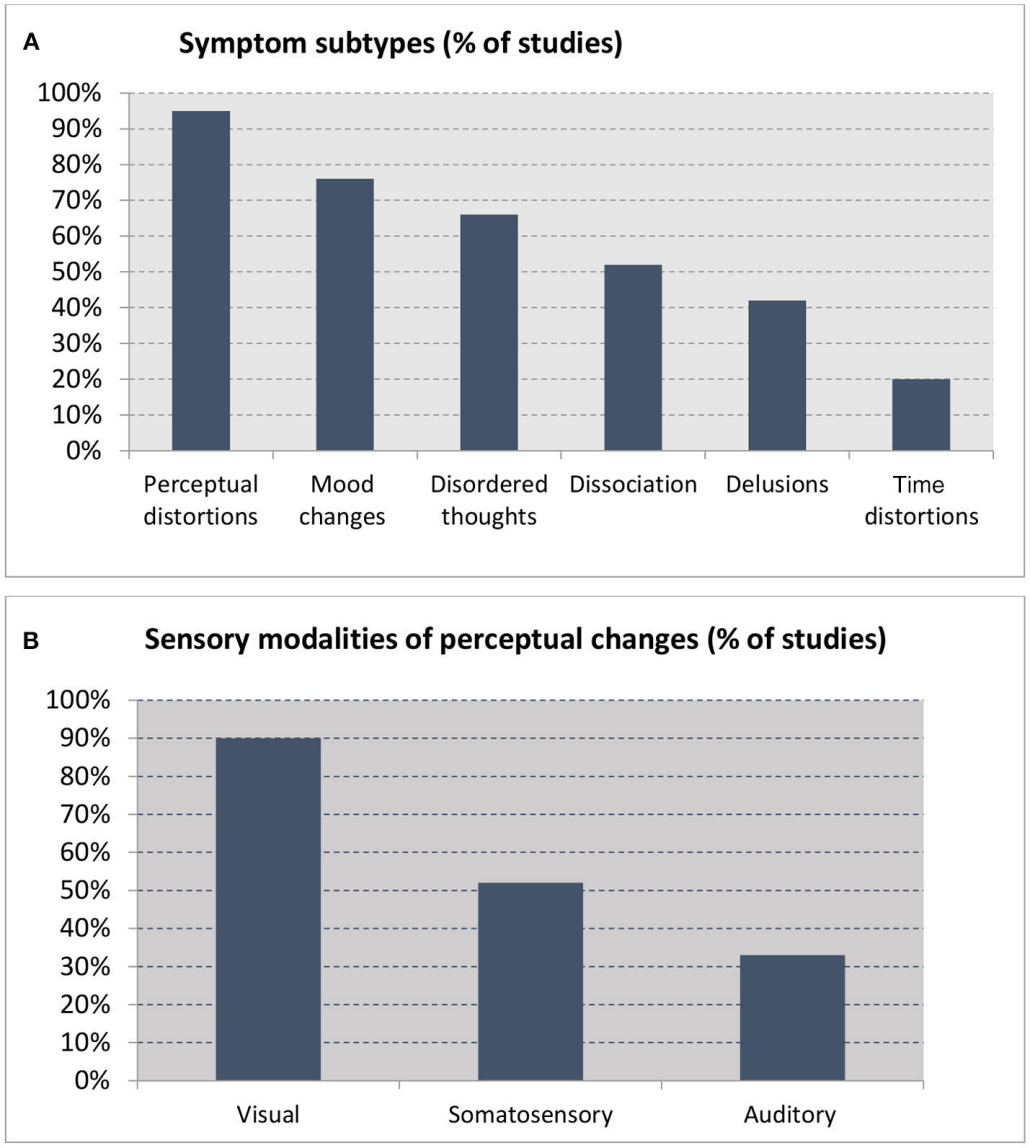
Percentage of studies reporting on **(A)** symptom subtypes (out of 21 studies) and **(B)** sensory modalities reported in studies reporting perceptual changes (*n* = 19).

The visual modality was the most commonly affected by sleep loss, as reported in all studies except one. Somatosensory changes were the second most common experience (reported in half of all the studies), followed by the auditory modality (a third of the studies) (see Appendix in Supplementary Material for descriptions) (Figure 2B). Symptoms included the following, in descending order of frequency:

*Visual experiences* (19 studies), which included a spectrum of symptoms, ranging from visual distortions, illusions, and hallucinations (Appendix A in Supplementary Material). These included visual *distortions (metamorphopsias)* (13 studies), referring to distortions of size (e.g., the room appearing larger), movement (e.g., stationary objects would appear to move), color (e.g., the floor becoming darker), contours (e.g., the forms of objects would lose their sharp angles), or duplications. These distortions were experienced intermittently, and elicited behavioral reactions ranging from surprise to irritation. *Visual illusions* (13 studies) comprised the transformation of common items (e.g., a switch, a fire alarm, a jacket) into other inanimate, but recognizable objects (e.g., a doll, a rock), or sometimes into animals or persons. Finally, 12 studies reported *visual hallucinations* which were generally transient and fleeting in nature, and most often of the simple type (e.g., indefinable substances growing from the floor) or the geometric type. Complex visual hallucinations (fully formed images) were reported in five studies, and involved the sudden appearance of animals, people or objects which were not really there. Some participants saw only halves of these hallucinated objects (“splitting”) (35, 36). In 10 studies, participants reported all three types of visual misperception (distortions, illusions, and hallucinations).

*Somatosensory experiences*, reported in 11 studies (Appendix B in Supplementary Material). These were bodily distortions (e.g., involving changes to the size of one's own body), as well as illusory sensations of movement (e.g., acceleration). There were also tactile hallucinations (e.g., the sensation of being touched), and temperature hallucinations (feeling heat or cold) (Appendix B in Supplementary Material). These changes were initially brief in nature, but they became increasingly persistent over time (and described as “creepy”), and acted upon with vigorous behavioral reactions.

*Auditory experiences*, reported in seven studies (Appendix C in Supplementary Material). Auditory distortions, described in all of these seven studies (Appendix C in Supplementary Material) were brief in duration, and included the mislocation of externally generated sounds, as well as changes in the quality of voices and other sounds (37). Participants also experienced hearing voices in the midst of other environmental sounds (“functional hallucinations”) (35), verbal auditory hallucinations of a simple type without affective content [e.g., a voice calling the participant's name in (34)], and nonverbal auditory hallucinations (e.g., dogs barking).

There were also reports of multimodal (i.e., compound or fused) hallucinations comprising two or more sensory modalities (28, 30). In four studies, hallucinations were “shared” and accepted as real by different participants (30, 35, 38, 39), two of which involved the collective hallucination of an “imaginary hat” (in (30), by 20% of 26 participants; and in (39), by all 12 participants).

### What other symptoms are elicited after sleep deprivation?

A range of other symptoms were reported, as follows (see Figure 2):

*Mood changes* (16 studies, 76%), which included aggression, anger, hostility, apathy, anxiety, and depression (9, 23, 26, 28–31, 34, 37, 38, 40–44).

*Disordered thoughts, confusion, and bizarre behavior* (14 studies, 66%), with studies commonly reporting confusion, difficulties with attention and concentration, fragmented thinking, and nonsensical speech. Participants described that their thoughts had become jumbled, and reported difficulties forming thoughts, finding words, and composing sentences (28, 31, 35–38). Memory loss was also a common feature, with participants forgetting names (34, 35, 37, 39, 41, 45). Motor incoordination, unsteadiness, and ataxia, comparable to intoxication behavior, were also reported.

*Dissociation and depersonalization* (11 studies, 52%), with participants experiencing a feeling of being separated from others, and estrangement (31, 33, 34, 37–39, 41). One participant reported, “I feel as if I'm not really all there. I am discontinuous. I feel discontinuous” (31). Another said, “I thought perhaps I might be somebody else, so I asked my buddies and they said I was just myself” (30). The sensations of splitting, being detached, and observing one's own body from a distance were also reported by some (39). These feelings of distance persisted until the end of the experiment (33).

*Delusions* (nine studies, 42%) (Appendix D in Supplementary Material) involved a range of classic delusional themes typically seen in schizophrenia-spectrum disorders, including primary delusions (44), delusions of control (36), persecution or paranoia (3, 28, 35, 39, 41), delusions of jealousy (41), delusions of grandeur (41, 43), and delusions of reduplication (40, 41). One participant “felt responsible for the Egyptian-Israeli conflict, and that a female secret agent in Florida was trying to get him to return to the Suez Canal” (41), and another imagined himself to be “on secret missions for the president” (32). Another “had the conviction that his fellow participants were plotting to kill him and were going to stab him in the back with a pen knife” (39). One participant (44) asserted that an article in the newspaper was “a sign that aliens wanted to take him to another world to create a new order.”

*Distortions in the sense of time* (four studies, 20%): Participants reported that time was a “hodgepodge” and “seemed to pass slowly” (30, 33, 37). As time without sleep increased, errors in time judgement occurred more frequently, and gross temporal disorientation was reported (30, 37).

### Do symptoms evolve or change over time as a function of increasing time spent awake?

**Figure 3:**
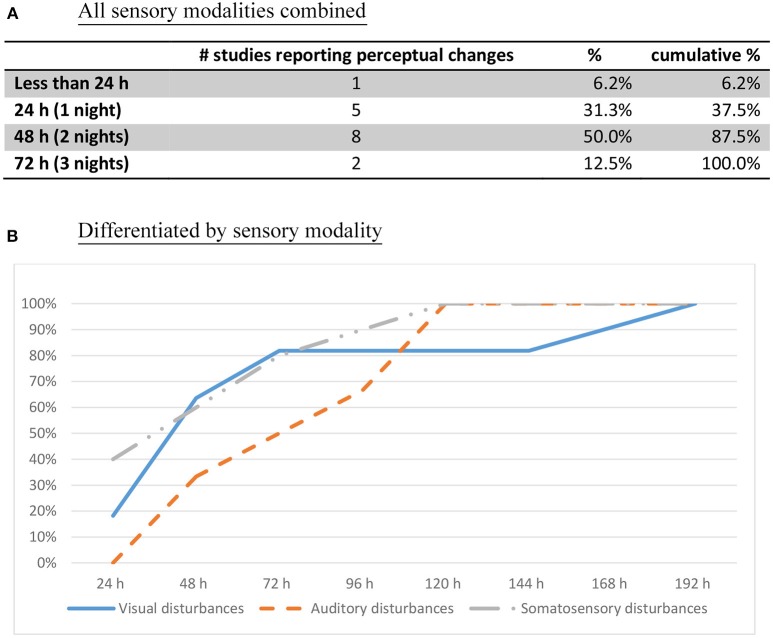
First onset of perceptual changes (cumulative percentage of studies reporting the time of perceptual change, *n* = 16). **(A)** All sensory modalities combined. **(B)** Differentiated by sensory modality.

**Figure 4:**
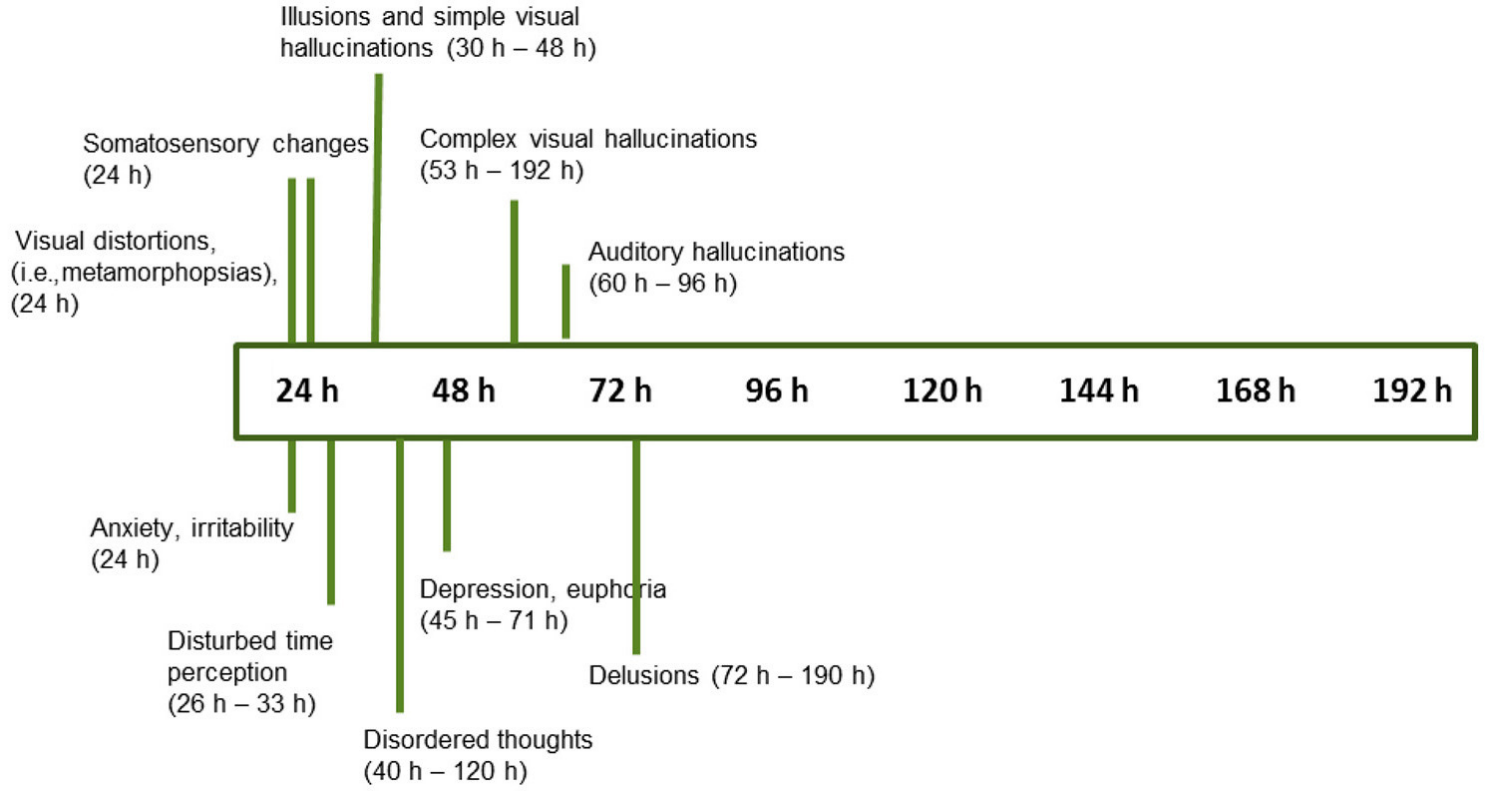
Progression of symptom onset as a function of wakefulness duration, with time range at which symptoms were first reported (*n* = 18 studies, see text for references).

We examined the time course of symptom development with increasing duration of sleep loss. The time at which symptoms were first elicited was extracted from each study. The results showed similar reports regarding the progression of symptoms with increasing time spent awake (Figures 3, 4). A number of observations can be made:

- Perceptual changes rarely make an appearance before 24 h have elapsed. Only after a whole night without sleep do these experiences start making an appearance (Figure 3A). After 48 h of sleep deprivation, perceptual distortions and hallucinations are reliably elicited (87.5% cumulative percentage of studies).
- Perceptual changes start with blurred vision and diplopia, with the visual complaint progressing gradually from distortions to illusions, and finally hallucinations (Figure 4, (18, 25, 26, 28–30, 32–38, 42, 45–47)].
- Misperceptions in different sensory modalities have different onset times. After one night without sleep, the progression starts with visual distortions (depth, size, and shape), and changes in the sense of body (18, 23, 25, 26, 33, 37), followed by visual illusions and simple hallucinations (30–48 h) (18, 28, 33, 35, 36, 42, 45, 46). After 50 h without sleep, there is a progression toward complex visual hallucinations, auditory hallucinations, and multimodal hallucinations (28–30, 32, 34–37, 42, 48) (Figure 4). These continue to increase gradually in complexity, severity, and persistence over time.
- Appraisals also undergo some changes. Initially, participants tend to question the veracity of the deceptive perceptual phenomena. With the passing of time and persistence of symptoms, there is a gradual acceptance that these events might be real, which precedes the appearance of full-blown delusional explanations.
- Disordered thoughts start to manifest by the second day, followed by delusions by the third day (Figure 4). These disturbances gradually but consistently increase in frequency over time, until the fifth day (sometimes called the “turning point”), characterized by a sudden deterioration of participants' mental health and the demonstration of acute psychotic symptoms with persistent hallucinations, delusions, and aggression (28, 29, 32, 35–37, 39, 40, 48). By that stage, delusions are elaborated and firmly held (35, 39).
- A host of other changes also occur progressively, beginning with depersonalization, dissociation, and temporal disorientation, beginning after 24–48 h of sleep loss, which increase in frequency over time (28, 33, 36, 38, 42).
- Mood changes include signs of anxiety and irritability within 24 h (23, 25, 26, 30, 31, 37), followed by depression, apathy alternating with euphoria, anger, and hostility within 45 h without sleep (28, 29, 32, 34, 37–42, 46).

### Do symptoms spontaneously resolve after a period of normal sleep?

The resolution of symptoms after a period of normal sleep was examined in 11 studies. Participants required approximately 50% of the total time they had been awake to recover from the deprivation period [e.g., 50 h of sleep to recover from 100 h of sleep deprivation; (34)].

Seven studies (*n* = 169 participants) reported full recovery after being allowed to sleep (32, 34, 35, 37, 38, 42, 45). One study described that the participant was “quite normal” after the first night of recovery (35), and another that some participants required several days with normal sleep to fully recover (34).

The other five studies described ongoing problems in some (but not all) participants, such as confusion, negative affect, mania, and delusional ideation, for days or even weeks after the experiments had been completed [(28, 33, 39, 41) *N* = 12; (36), *N* = 3]. It should be noted that two of the studies included a participant with a documented history of mental health problems (both *N* = 1) (28, 41).

## Discussion

This study reviewed sleep-deprivation experiments where duration of sleep loss ranged from one to 11 nights (24–264 h) (*n* = 735). The findings showed a remarkably broad range of perceptual anomalies and other symptom experiences. The visual modality was the most prominently affected, but other symptoms included bodily sensations, mood changes, disordered thoughts, dissociation, delusions, and temporal disorientation. Importantly, symptoms appeared in a time-dependent way, and rapidly progressed with increasing time awake. Various studies suggested that symptoms largely resolve after recovery sleep, although some participants showed ongoing effects days or even weeks after the study had ended. We address the findings in turn, before speculating about the biological processes which might be involved.

### The impact of sleep deprivation on perceptual distortions, hallucinations and other symptoms

Perceptual distortions and hallucinations were reliably elicited in 20 out of 21 studies reviewed here. These findings support the results of population studies which show that sleep loss is tightly linked with hallucinations [e.g., (19, 21, 22)], but also demonstrate that hallucinations can be a direct consequence of chronic sleep deprivation.

Detailed examination of the symptoms described revealed that the visual modality was the most prominently and consistently affected (reported in 90% of the studies), followed by the somatosensory and auditory modalities (52 and 33%, respectively). Symptoms included a spectrum of phenomena ranging from visual distortions (color, size, depth, and distance), illusions (misidentification of common objects or sounds), and finally hallucinations (simple, complex and compound), which developed in a time-dependent way. In most cases, these perceptual phenomena were experienced as vivid and real, and hardly amenable to volitional control.

All studies reviewed here had a total-sleep-deprivation design, except for two, where intermittent sleep had been allowed (akin to a sleep-restriction design). It is interesting that the one study which failed to report perceptual changes involved one such design, where a brief sleep period had been allowed in medical interns working a 32-h work shift (<6% of the total duration of sleep loss). A number of reasons may have contributed to a lack of symptoms in that group, including the short period of sleep which may have served to reduce the impact of sleep loss on brain functions; an attentional focus on tasks of high importance which may have acted to maintain perceptual stability [e.g., (49)]; or a negative response bias because of the risk to be perceived as unprofessional or unfit for duty.

Other experiences reported after sleep loss included mood changes (76%), disordered thoughts and memory loss (66%), dissociation and depersonalization (52%), delusions (40%), and distortions in the sense of time (20%). These symptoms were largely inconstant, and came in waves. These findings suggest that the effects of sleep deprivation can be pervasive and wide-ranging in affecting all areas of thought and functioning, and are in line with previous findings demonstrating that prolonged sleep loss adversely affects cognitive functions including attention, concentration and memory (50–52), negative mood (including depression), fear, and tension (53, 54).

### Symptoms evolve toward psychosis with increasing time spent awake

One of the most intriguing findings was the evolution of symptoms over time, together with the order in which they appeared, which was remarkably similar amongst studies. There were no or few changes in the first 24 h, and still rather mild perceptual anomalies accompanied by anxiety and irritability after the first night without sleep. After 48 h, marked psychological symptoms and perceptual disorders were described.

The perceptual changes seemed follow a more or less fixed development from distortions to illusions, and finally hallucinations, beginning with the visual modality, followed by somatosensory changes, and finally changes in the auditory modality. By the third day without sleep, all three sensory modalities were affected. Appraisals also changed over time, from a questioning stance to full acceptance as symptoms persisted over time.

The final effects to appear were psychotic symptoms such as thought disorder, and delusions. After 5 days, a clinical picture resembling that of acute psychosis or toxic delirium appeared. The finding that sleep deprivation can apparently produce symptoms of acute psychosis in healthy individuals adds to the evidence linking sleep and psychosis. In support, various studies show that prolonged sleep loss is both a precursor and precipitant to psychosis (8, 10–12).

### Resolution of symptoms after restorative sleep

After a return to normal sleep, the great majority of studies reported complete recovery without any further complications or symptoms (82%). In Tyler's (43) study of sleep deprivation in 350 male army recruits (112 h without sleep), no residual mental health problems were reported in any of the participants. In five studies, individual participants continued to exhibit residual symptoms (e.g., confusion, negative mood, delusional beliefs) for days or even weeks after the experiment. Pre-existing mental health conditions were cited as a likely cause. The two participants in the studies by Brauchi and West (41) and Katz and Landis (28) subsequently revealed a documented history of mental illness. A limitation of all studies includes varied follow-up periods (ranging between 1 day and 6 months after the experiment), and that follow-up assessments did not include detailed psychological assessments. Overall, there is insufficient data to determine the optimal duration of recovery sleep, and to understand the factors that better assist people with their return to normal baseline.

Individual differences in the resilience to the effects of sleep deprivation support the vulnerability-stress model, according to which some individuals with a prior vulnerability are at risk of developing mental health issues after exposure to a stressor (55). Individuals with a psychiatric illness are also at a greater risk of relapse after a period of stress, with sleep deprivation being a reliable stressor capable of eliciting rapid decompensation (56). Hypothesized mechanisms involve the hypothalamo-pituitary-adrenal (HPA) axis, and elevated cortisol levels (57), along with pro-inflammatory cytokines (58), which act to increase reactivity to stress. Interestingly, these biological markers have also been implicated in some sleep-deprivation studies (59–61), although a link to outcomes remains to be shown.

### Similarities and differences with psychiatric disorders and other conditions

This study shows that going without sleep for 2 days can produce some powerful hallucinatory experiences which can be mistaken for veridical perceptions, and over which participants have little control. An examination of the phenomenological features of these experiences reveals a very unique profile which has not been documented before, nor readily observed in other conditions or disorders.

In the early stages of sleep deprivation (2–3 days), visual phenomena are more similar to visual percepts reported in eye disease (e.g., Charles Bonnet syndrome) and Parkinson's disease (PD) than to psychotic disorders such as schizophrenia. Similar to eye disease and PD, individuals report simple percepts (flashes, lights, dots) and complex visual hallucinations where the contents are affectively neutral and rarely perceived as frightening. Unlike these conditions however, sleep deprivation features a range of somatic and tactile sensations, as well as metamorphopsias and other phenomena which are considered rare, such as the sudden appearance and disappearance of only halves of objects (“splitting”), multisensory (“fused”) hallucinations, and collective (“shared”) hallucinations, which differentiates them from other conditions.

After 3 or 4 days, and with increasing time awake, some instances of auditory hallucination were reported. While some features are reminiscent of psychosis (mistaken for veridical perceptions and interpreted as symbolic), these were isolated and rare events, lacking the complexity and language sophistication of the voices described by individuals diagnosed with schizophrenia. The accompanying symptoms of thought disorder and delusion, however, resembled those observed in psychosis, although it is not clear whether these were persistent, or instead sporadic and intermittent.

We also wondered whether participants might actually have experienced hypnagogia or perhaps even dream-intrusion phenomena, given the resemblance to percepts often reported during the transition from wake to sleep (hypnagogic hallucinations) and sleep to wake (hypnopompic hallucinations). Some symptoms during sleep deprivation are indeed supportive of a continuation of dream sequences (28, 30, 33, 35, 37, 39, 42). As remarked about one participant, “*Soon afterwards, he kissed the EEG paper. When asked about this, he said he must have been dreaming about his girlfriend*” (35). Nonetheless, there are also important differences. Dreams tend to follow a narrative structure and the dreamer is immersed as the protagonist (62, 63), which is a feature which appears to be lacking in most sleep-deprivation experiences. Moreover, typical hypnagogic phenomena include light flashes, “faces in the dark,” and ruminations or continuations of previous conversations (64–66) which are unlike those experienced during sleep deprivation. Finally, participants themselves also judged their visual and other perceptual experiences to be distinct from dreams, so it would seem unlikely that they consisted entirely of hypnagogia or dream phenomena.

### Hypotheses regarding the brain processes involved

The gradual development of symptoms starting with blurred vision and diplopia, progressing to visual distortions and illusions, and finally hallucinations in multiple sensory modalities, points to a gradually weakening perceptual system. Initially, the visual network appears gradually compromised, with—subsequently—similar effects in the somatosensory and auditory modalities and cognitive domains.

Symptoms starting with visual problems perhaps speak to an early involvement of the occipital cortex. In support, neuroimaging studies describe the visual-sensory and visual-attention areas as the first domains to be affected by sleep deprivation. These have been shown to cause disturbances in the quality of perceptual representations (49, 67, 68). In addition, migraine auras, which produce metamorphopsias of the type reported here (69), also show visual distortions from cortical spreading mechanisms starting in the occipital cortex moving toward prefrontal areas of the brain (70). It is worth noting that the proposal of a gradual change in brain processes in a posterior-anterior direction stands in contrast to neurophysiological findings, which have observed the direction of neuronal activity during pre-sleep and early sleep-deprivation periods as starting in anterior areas before spreading to posterior ones (71, 72). The lack of correlations between neuroimaging signals and basic electrophysiological functions, however, is not uncommon (73), since these methods of investigation provide different levels of explanation, as well as different temporal and spatial resolutions.

The psychopathological symptoms elicited by sleep deprivation are in line with studies demonstrating the severe effects of sleep loss on cortical and subcortical functions, health, and functioning (2, 72). The process may involve a destabilization of central functions causing profound changes in brain functions in a dose-dependent manner (74, 75). Although the exact mechanisms of extreme sleep deprivation are still in need of further elucidation, the involvement of perception areas is undisputed, as is the involvement of prefrontal areas. It has been suggested that functions involving the medial frontal cortex and thalamic activations falter with increased duration of sleep loss, causing mental “lapses” and reductions in alertness (2, 76). Such lapses may impact on perceptions because the brain increasingly focuses inwards, and is becoming less constrained by input from the external environment. Through such changes in signal-to-noise ratio, and decreases in error monitoring and error detection (77), the key mechanisms which are traditionally deemed to support hallucinatory activity are enabled (78, 79).

### Neurotransmitter action

The underlying biological mechanism for these perceptual changes may be neuronal instability (80) or a related defect in neural transmission. Because of the prominent impact of sleep deprivation on visual processes, we may speculate on the role of cholinergic (ACh) transmission because of its role in the visual sensory modality. In the absence of external perceptual signals, the cholinergic system has the potential to spontaneously produce excitatory signals to the sensory areas, along with changes in signal-to-noise ratio during information processing (81, 82), which are two key mechanisms implicated in hypnagogia and dreams and which would support the involvement of occipito-frontal acetylcholine depletion mechanisms. Furthermore, displays of myasthenia-like weakness of voluntary muscles during sleep deprivation, including an inability to raise the eyelids, have been linked to cholinergic depletion (83).

The symptoms' similarities with psychosis may also hint at the involvement of dopaminergic, noradrenergic, and serotonergic processes (84). Support for a role of dopamine comes from animal studies, which show that sleep deprivation produces a state of supersensitivity of post-synaptic dopamine receptors (85, 86). While direct evidence for dopamine involvement in sleep deprivation in humans is lacking, ACh and nicotinic receptors (both with links with occipital cortex) (87) are key mediators of dopamine (88), with a synaptic balance existing between ACh and dopamine in the striatum, and nicotinic receptors located on dopamine nerve terminals (89). Furthermore, the involvement of noradrenaline, serotonin, and ACh plays an important role in sleep and wake processes (90, 98).

Another candidate mechanism for the mediation of perceptual and other mental symptoms in sleep deprivation is central chromatolysis, an intracellular degenerational process due to fatigue and stress, which is characterized by a dissolution of Nissl bodies. Chromatolysis is not only a robust intracellular effect of fatigue and stress, it is considered one of the three known principal mechanisms of cell death (91). Neurons which are selectively vulnerable to damage are found in the cerebral cortex in regions responsible for sensory perception and executive functions. In animal studies, ischemic events produce cell changes in these regions in the temporal range of 24–72 h, which is the window during which the build-up to full-blown sleep deprivation effects takes place (92). Additional support for the chromatolysis hypothesis comes from the symptoms of pellagra, a disorder characterized by central chromatolysis. Pellagra is a nutritional disorder with niacin deficiency constituting the central mechanism (93, 94). In addition to classical symptoms of dermatitis, diarrhea, and dementia, the mental health symptoms can be mistaken for schizophrenia (95), and are similar to those in prolonged sleep loss. Similarly, prolonged sleep deprivation in rats and other animals can cause skin lesions characteristic of pellagra (96, 97).

### Limitations

Several limitations apply to the current study. First, the entire analysis was retrospective in nature. Moreover, as current ethical guidelines prevent replication studies of prolonged sleep duration, new reference material cannot be collected. Secondly, methodological limitations of the original studies included broad variations between study protocols, and a lack of systematic utilization of validated scales, which may have contributed to inconsistencies in symptoms being reported. However, log books and experimenter notes revealed mental events previously unrecorded (e.g., metamorphopsias)—which might have remained unexposed if quantitative measurements had been used. In addition, despite these variations in protocols, the results were surprisingly consistent in showing perceptual and other mental changes in a fixed temporal order. Thirdly, our interpretation of the findings may well have been confounded by comorbid stress, a major confounder in studies such as these. Because of that, existing models discussed above are in need of further elaboration in the context of all the neurobiological consequences of sleep loss, which are manifold, complex, and still only partially understood.

## Conclusions

Our examination of 21 studies involving sleep deprivation of 24 h to 11 days shows that sleep loss can be a direct cause of prominent hallucinations and other misperceptions, as well as mood changes, distorted thinking, delusions, depersonalization, and time distortions. These symptoms start to appear after 24 h without sleep, and undergo increases in intensity and frequency until sudden deterioration by the fifth day, characterized by acute psychosis. Insufficient evidence exists regarding the period of sleep needed to make a full recovery, and about the factors which are responsible for ongoing mental health problems some weeks after the completion of the experiment.

## Author contributions

FW contributed to the conception and design of the work, and to the acquisition, analysis, and interpretation of data for the work, drafted and revised the work, gave final approval for the final version to be published, and agreed to be accountable for all aspects of the work in ensuring that questions related to the accuracy or integrity of any part of the work are appropriately investigated and resolved. VC contributed to the acquisition, analysis, and interpretation of data for the work, revised the work, gave final approval for the final version to be published, and agreed to be accountable for all aspects of the work in ensuring that questions related to the accuracy or integrity of any part of the work are appropriately investigated and resolved. AA contributed to the acquisition, analysis, and interpretation of data for the work, revised the work, gave final approval for the final version to be published, and agreed to be accountable for all aspects of the work in ensuring that questions related to the accuracy or integrity of any part of the work are appropriately investigated and resolved. JDB contributed to the conception and design of the work, and to the interpretation of data for the work, drafted and revised the work, gave final approval for the final version to be published, and agreed to be accountable for all aspects of the work in ensuring that questions related to the accuracy or integrity of any part of the work are appropriately investigated and resolved.

### Conflict of interest statement

The authors declare that the research was conducted in the absence of any commercial or financial relationships that could be construed as a potential conflict of interest.
