# Supplementary material for: Severe Sleep Deprivation Causes Hallucinations and a Gradual Progression Toward Psychosis With Increasing Time Awake
Source: Front Psychiatry. 2018 Jul 10;9:303. doi: 10.3389/fpsyt.2018.00303 (PMC6048360; doi:10.3389/fpsyt.2018.00303)
Supplement: Supplementary file 1 [file Data_Sheet_1.docx]

**Supplementary material with definitions (after Blom, 2010*)**

*Blom, J.D. (2010). *A Dictionary of Hallucinations*. New York, NY: Springer.

**Appendix A: Visual phenomena**

| ***Metamorphopsias*** | |
| --- | --- |
| *Dysmorphopsia* | Definition: Lines and contours appear to be wavy |
|  | - The legs of a table occasionally quivered (Bliss et al., 1959) - A nurse's cap would undulate and twist (Morris et al., 1960) - The definite form of the images was replaced by undulating, wavy, irregular borders without sharp angles (Malmo & Surwillo, 1960) - The floor seems wavy (Williams et al.,1962) - Wallpaper patterns seemed to move and flow (Berger & Oswald, 1962) - Dark squares of the floor tile seemed to pulsate (Bliss et al., 1959) |
| *Kinetopsia* | Definition: Illusory movement |
|  | - Monitor was seen to be swimming around (Babkoff et al., 1989) - Crumbs on the table-cloth running about like insects (Berger & Oswald, 1962) - The Coke machine was moving up and down (Bliss et al., 1959) - Spots on the floor seemed to move about (Morris et al., 1960) - Stationary objects would appear to move suddenly (Morris et al., 1960) |
| *Micropsia, microtelepsia, macropsia, microproxiopia* | Definition: Objects appearing smaller (micropsia) and further away than they are (microtelepsia), or objects appearing larger (macropsia) or closer by than they are (microproxiopia) |
|  | - I thought to myself "Gad, it's completely changed—much smaller and different" (Kollar et al., 1966) - People seemed to be in the distance (Edwards, 1941) - The men's room seemed larger and the floor higher (Bliss et al., 1959) - His hands seemed to be larger (Bliss et al., 1959) - This layer was a foot above the floor and parallel with it (Patrick & Gilbert, 1896) - It looked as though the room were on a second story, and it appeared to him that it was necessary to climb a step to get to it from the hall (Bliss et al., 1959) - Squares of the floor would become larger, with every pulsation (Bliss et al., 1959) |
| *Achromatopsia* | Definition: Inability or diminished ability to perceive colour |
|  | - The dark squares of the floor would become darker (Bliss et al., 1959) - The colour of the chairs seems to change (Morris et al., 1960) - The lights seem to flicker (Williams et al.,1962) |
| *Polyopia* | Definition: Multiple identical copies of a single image |
|  | - Several soap dishes (Katz & Landis, 1935) |
| ***Illusions*** | |
| *Illusions* | Definition: A real stimulus which is misperceived or misinterpreted   - The switches on the control panel seen as kewpie dolls (Anderson & Gorfein, 1964) - The research assistant was seen as a fire hydrant (Babkoff et al., 1989) - An old lady sticking her head out from the room. “*She seemed to have grey hair, which was rather frizzy. I stopped to look again. She still had her head showing, and it did not move. It wasn't until about 10 yards from her that I realized it was a fire alarm box*.” (Bliss et al., 1959) - The subject saw a mannequin standing by a refrigerator and thought that it was a woman waiting to get into her car. He actually opened the refrigerator door to assist the "woman" in out of the rain (Brauchi & West, 1959) - The subject saw gloves on a girl's hand when the girl had on no gloves (Edwards, 1941) - The subject walked to a desk which he mistook for a drinking fountain (Katz & Landis, 1935) - A transatlantic whale with a square face in the crystal (Katz & Landis, 1935) - The knobs became people (Malmo & Surwillo, 1960) - That black mark looked like it was changing into different rock formations (Morris et al., 1960) - “I saw hair in my milk. The others said there wasn't any, but I still felt there was and would not drink it.” (Morris et al., 1960) |
| ***Hallucinations*** | |
| *Hallucinations* | Definition: a perception, experienced while awake, without a corresponding stimulus in the outside world   - The monitor was seen to be growing hair (Babkoff et al., 1989) - The decaying corpse of Barbara (assistant) interfered with vision during the administration of the mood scale (Babkoff et al., 1989) - Swirling vapour, cobwebs or shimmering bubbles seemed to cover the floor, their hands, or the table (Berger & Oswald, 1962) - He saw a roll of luminous chicken wire on the floor (Bliss et al., 1959) - Geometric designs of great complexity (Luby et al., 1962) - All subjects reported smoke and haze arising from objects *(*Kollar et al., 1966) - Subject saw old women peering at them in broad daylight (Berger & Oswald, 1962). - One reported that the old woman appeared to be talking about him (Berger & Oswald, 1962) - Indistinct hallucinations, halves of large objects. There were patterns of them: rows and squares, which were interesting but annoying (Malmo & Surwillo, 1960) - Woman’s body would vanish as the subject would get closer (the body before the face) and then return (Berger & Oswald, 1962) - Floor of the laboratory seemed to be covered by a layer of shimmering water (Bliss et al., 1959) - Fine smoke and jets of water coming from the linoleum floor cover (Bliss et al., 1959) - The air was full of these dancing particles which developed into swarms of little bodies like gnats, but coloured red, purple, or black (Patrick & Gilbert, 1896) - Fog, mist, or smoke issuing from under doors or from the walls (Luby et al., 1962) - Fuzz around the bottle (Morris et al., 1960) - Steam rising from the floor (Morris et al., 1960) - A person across the table appearing and disappearing from vision several times (Edwards, 1941) - Cobwebs covering his hands and face while he was washing up, and he could both see and feel them (Morris et al., 1960). - Handwriting appeared on a jacket (Berger & Oswald, 1962) - Two walked into a wall because they had hallucinated an open door *(*Kollar et al., 1966) - The subject saw a cottage by a beach, then other houses. He then saw a man trying to burglarize one of the houses (Katz & Landis, 1935) - He saw very clearly two boats sailing across together, then a missionary in a roast pot with his knees hanging over the edge. The image turned into an old man in a bathtub with his knees hanging over (Katz & Landis, 1935) - He saw a boy with cheeks puffed out giving a Bronx cheer (Katz & Landis, 1935) - The hallucinated figures were two-dimensional ones, projected some distance from the eyes as if on a movie screen (Malmo & Surwillo, 1960) - The subject would climb upon a chair to brush them off (Patrick & Gilbert, 1896) - Tried to touch an imaginary fly on the table with his finger (Patrick & Gilbert, 1896) |

| **Appendix B: Somatosensory phenomena** | |
| --- | --- |
| ***Distortions*** | |
| *Hypoaesthesia, anaesthesia* | Definition: diminished perception, absence of perception   - Felt his body/knee was numb and insulated (Bliss et al., 1959) - Unable to distinguish hot and cold when they took baths; reported no feeling in the skin; sides of head had no feeling; skin “felt dead”; numbness across the forehead; no feeling in finger tips (Edwards, 1941) - Feelings of numbness (Luby et al., 1962) |
| *Hyperaesthesia* | Definition: exaggerated type of perception   - "Creepy" hypersensitivity all over the subject’s skin that was constant and lasted throughout the final 48 hours of sleep deprivation (Bliss et al., 1959) |
| *Micro- or macro-somatognosia* | Definition: experiencing (parts of) one’s body as smaller or larger   - Change in perception of size of own body (Cappon & Banks, 1960) - Feeling that body was weightless (Cappon & Banks, 1960) |
| *Paraesthesia* | Definition: altered somatic sensation, typically characterized by numbness, tingling, prickling or ‘pins and needles’   - Peculiar feelings in his hands. A warm, tired feeling in his arms and legs. Felt a tight sensation over his face (Bliss et al., 1959) - Peculiar, tingling feeling all over body (Edwards, 1941) - Feeling light as if one were walking on things that were soft or feeling that the pavement was mushy (Edwards, 1941) |
| ***Illusions / Hallucinations*** | |
| *Kinesthetic hallucination, space-motion hallucination* | Definition: Illusory movement of the body and/or its surroundings   - Feeling that body was accelerated (Cappon & Banks, 1960) - Feeling of moving (Malmo & Surwillo, 1960) - A shaking sensation that left the subject feeling there had been an earthquake (Morris et al., 1960) |
| *Slow-motion hallucination* | Definition: illusory slowing down of one’s body   - Muscles felt stiff and sluggish (Bliss et al., 1959) |
| *Automatism* | Definition: unconsciously generated sensory and/or motor phenomena   - One was walking down the hall when his left heel seemed to raise by itself (Bliss et al., 1959) |
| *Somaesthetic hallucination* | Definition: hallucination experienced in the somatosensory modality   - A "funny," electrical, cold sensation shot up the back of his leg. A fleeting flush over his skin (Bliss et al., 1959) - Feeling of objects in eye (Edwards, 1941) - Felt a peculiar “buzzing in the head” (Kleitman, 1923) |
| Tactile hallucinations | Definition: hallucination of touch   - Feeling of a band pressing on the head (Edwards, 1941) - The experience of wearing a hat was reported by every participant in the project (Luby et al., 1962) - He felt cobwebs covering his hands and face while he was washing up, and he could both see and feel them (Patrick & Gilbert, 1896) - 20% reported the "hat illusion” (a band of pressure around the head). Subjects were often observed making repeated efforts to remove the non-existent hat (Morris et al., 1960) |

| **Appendix C: Auditory phenomena** | |
| --- | --- |
| ***Distortions*** |  |
| *Hyperacusis* | Definition: hypersensitivity to sound   - Subjects reported that loud sounds seemed harsh and disrupting, or that people’s voices seemed distant (Bliss et al., 1959) |
| ***Illusions / Hallucinations*** | |
| *Auditory illusion* | Definition: a sound that seems louder or softer, fainter or more distinct, nearer or farther   - One noticed that he could watch his companions' mouths as they talked next to him, yet feel their voices were coming from the next room (Bliss et al., 1959) - "Their voices seemed to come from somewhere else." (Bliss et al., 1959) - Hearing sounds seemingly at a considerable distance. Voices of those present seemed to be far away (Edwards, 1941) |
|  | - Heard different discrete tones from switching the knob around (Malmo & Surwillo, 1960) |
| *Auditory hallucination* | - After 96 hours of sleep deprivation, hallucinatory voices while a water tap was running (Berger & Oswald, 1962) - ‘Transient auditory and visual hallucinations occurred increasingly in both subjects at various times after the fourth day’ (Brauchi & West, 1959) - Heard housemother call her when she was not there (Edwards, 1941) |

**Appendix D: Delusions**

| Paranoid delusion  Delusion of reference | - A test person described one of the investigators as an "exquisitor", which meant an inquisitor able to inflict exquisite pain. He intended to hire a bodyguard to protect him from "them”. He believed he must have been given a hallucinatory drug. He believed that an investigator was taking another subject away to lock him up after first hypnotizing him (Berger & Oswald, 1962) - When playing a word game and being given the word "train", he replied "Glasgow", for it instantly dawned on him that another subject must be about to be locked up for having been responsible for the fires on the Glasgow electric trains withdrawn from service some months before (Berger & Oswald, 1962) - He developed the feeling that he was being punished by being made to stay awake. Then he believed that a female secret agent in Florida was trying to get him to return to the Suez Canal zone (Brauchi & West, 1959) - He formulated a delusional system, attributing persecutory intent to one of the experimenters. He became more and more certain that this experimenter was personally interested in making life disagreeable for him (Katz & Landis, 1935) - He had the conviction that his car had been stolen, that his fellow participants in the project were plotting to kill him, and that one attendant was going to stab him in the back with a pen knife. Strongly motivated by these fears, he attempted to protect himself by calling his wife to warn her that he might not survive, telling her to contact the “proper authorities” (Luby et al., 1962) - He thought that he noted some connection between the disappearance of the hallucinations and "disturbances" in the control room where the experimenters were operating the equipment. (Malmo & Surwillo, 1960) |
| --- | --- |
| *Delusion of* *grandeur* | - He felt that he and his competitor belonged to a secret club of non-sleepers. He also developed the delusion that he was responsible for the Egyptian-Israeli conflict (Brauchi & West, 1959) - This subject imagined himself on secret missions for the President (Tyler, 1947) - He believed that he was the Messiah and that he had the task of creating world peace |
| *R****eduplicative paramnesia*** | - He reported that he thought his broadcasting booth was atop a hotel in a city some 50 miles away ***(***Anderson & Gorfein, 1964) - During the fourth day and subsequently the patient thought that various parts of the equipment were in different cities (Brauchi & West, 1959) |
